# Effect of growth hormone administration on ameliorating pregnancy outcome in women with advanced maternal age and exploration of its optimized utilization

**DOI:** 10.3389/fendo.2023.1270897

**Published:** 2023-10-25

**Authors:** Qihui Feng, Yanbin Wang, Hongjing Han, Huan Shen

**Affiliations:** Reproductive Medical Center, Department of Obstetrics and Gynecology, Peking University People’s Hospital, Beijing, China

**Keywords:** advanced maternal age, growth hormone, dosage regimen, *in vitro* fertilization, pregnancy outcome

## Abstract

**Background:**

Age-related fertility decay is a great challenge for clinicians. Growth hormone (GH) supplementation has been studied as an adjuvant since late 1980s. However, it has not come to a consensus on the GH administration due to the ambiguous efficacy among studies with different enrolled population and dosage regime.

**Methods:**

A self-controlled retrospective study was conducted on women with advanced maternal age who underwent at least a previous cycle without GH (GH−) and a subsequent cycle with GH co-treatment (GH+). The ovarian stimulation parameters and outcomes were compared between the two cycles and logistical analysis was applied to further explore the association between GH administration protocol as well as other clinical parameters and cumulative live birth in GH+cycle.

**Results:**

A total of 150 women aged 35-43 were included. The number of oocytes retrieved, MII oocytes, 2PNs, transferrable embryos and good-quality embryos in GH+ significantly increased (*p* < 0.001). The proportion of cycles with no transferrable embryos was significantly reduced in GH+ cycle compared with previous GH− cycle (3 *vs.* 32; *p* < 0.001). GH co-treatment cycles showed significantly higher clinical pregnancy rates (43.75% *vs.* 6.06%; 38.35% *vs.* 12.04%, *p* < 0.001), live birth rates (29.17% *vs.* 0; 27.07% *vs.* 0, *p* < 0.001) in both fresh and frozen-thawed embryo transfer cycle. Cumulative live birth rate of the GH+ cycle reached 33.33%. Use of GH prior to Gn stimulation and lasting until the hCG day seemed to achieve a higher successful live birth rate (OR 2.312, 95%CI 1.074-5.163, *p*=0.032).

**Conclusion:**

GH supplementation could ameliorate pregnancy outcome in women with advanced maternal age. Dosage regimen of long-term pretreatment prior to Gn stimulation (4 IU every other day) and 4 IU per day until hCG day may of greater efficacy compared with concurrent administration with Gn. Additionally, it’s worthy of exploring whether an individualized dosage regimen based on the IGF or IGFBP level of patient would be more reasonable and effective. More well-designed prospective trials with large sample size and fundamental experiments on the mechanism are required to testify findings above.

## 1 Introduction

There has been an increasing proportion of women with advanced maternal age (AMA) in the assisted reproductive technology (ART). Due to the decline in quantity and quality of oocytes as well as the higher aneuploidy rates (1), age-related fertility decay remains to be a great challenge for fertility specialists, with no clear effective remedies to counteract (2).

Growth hormone (GH), a peptide hormone mainly secreted by adenohypophysis cells and known to play a vital role in cell growth, development and metabolism in multiple targeted tissues, has been demonstrated probably to be involved in folliculogenesis, steroidogenesis, oocyte maturation, ovulation, oocyte quality and ovarian response to gonadotropins (3–5) by directly acting on growth hormone receptor (GHR) (6) or insulin-like growth factor (IGF)- mediated (7, 8) as well. Since Homburg et al. firstly explored the effect of GH supplementation on augmenting the ovarian response to gonadotropin during controlled ovarian hyperstimulation (COH) in women relatively resistant to human menopausal gonadotropin (hMG) therapy since the 1980s (9–11), more and more studies have been implemented with different administration protocols under various clinical background, such as advanced maternal age (12, 13), poor ovarian response (14–19), polycystic ovarian syndrome (20) or repeated implantation failure (21, 22) et al.

Despite the gained evidence on the utilization of GH in ART, there are still many controversies on this issue, such as the target population, dosage regimen, underlying mechanism and so on. Here, a retrospective self-controlled analysis was conducted among women aged 35-43 with at least a previous cycle without GH and a subsequent cycle with GH co-treatment to investigate the effect of growth hormone co-treatment on the embryo quality and pregnancy outcome and explore the possible factors influencing the outcome following GH administration to optimize its utilization.

## 2 Materials and methods

### 2.1 Participants

A retrospective, self-controlled study was conducted among 150 women with advanced maternal age who underwent at least a previous cycle without GH supplementation (GH−) and a subsequent cycle with GH co-treatment (GH+) from September 2016 to June 2022 in Reproductive medical center of Peking University People’s Hospital.

### 2.2 Clinical management

There were no strict limitations on the ovarian stimulation protocols, which were applied by the attending clinician based on age, ovarian reserve, complications and ovarian response to the previous cycle. When ≥ 2 leading follicles reached 18mm in diameter, final oocyte maturation was triggered by the administration of 250mg of recombinant hCG (Ovidrel; Merck Serono, Geneva, Switzerland) alone, or 0.2mg of triptorelin (Ferring International Center, SaintPrex, Switzerland) plus 2000 IU of hCG (Livzon, Zhuhai, China). Oocyte retrieval was performed under transvaginal ultrasonography 35-37 h later. Other clinical procedure protocols of embryo culture, embryo transfer, luteal phase support was following the routine clinical criteria of our center (23). There were three GH (Saizen; Changchun GeneScience, Changchun, China) administration protocols among the included participants (1): 4 IU every other day from previous menstrual cycle day 1-3 or the day of gonadotropin-releasing hormone agonist (GnRH-a) injection, then changed into 4 IU daily from the initiation of ovarian stimulation and lasting until hCG day (4 IU QOD – 4IU QD) (2); 4 IU every other day from previous menstrual cycle until the initiation of Gn (4 IU QOD) (3); 4IU daily form the initiation day of ovarian stimulation to hCG day (4IU QD).

### 2.3 Outcome measures

The primary outcome was the cumulative live birth rate per oocyte retrieval cycle. Here, based on the characteristics of the enrolled patients, we defined a cumulative live birth rate per oocyte retrieval cycle as the number of women who achieved their first live birth (including the fresh embryo transfer and frozen-thawed embryo transfer of the embryos collected in this cycle up to now) divided by the number of women who attempted stimulation. Day-3 good-quality embryos referred to the embryos reached 7-10 cell stage, equal or slightly unequal blastomeres and ≤15% fragmentation on day 3. Biochemical pregnancy was defined as serum β-hCG > 50U/L on day 12 after blastocyst transplantation or day 14 after the cleavage stage embryo transplantation. Clinical pregnancy was defined as a pregnancy confirmed by ultrasound visualization of the gestational sac 4-5 weeks after embryo transfer. Miscarriage rate was defined as the number of cases with pregnancy loss within 28 weeks of gestation starting from the day of oocyte fertilization divided by the number of clinical pregnancies.

### 2.4 Statistical analysis

Categorical variables were presented as number and percentage. Continuous variables normally distributed were presented as mean ± standard deviation (SD) while as median (interquartile range, IQR) if not normally distributed. Comparison of continuous variables between the two groups was performed using paired t-test (normally distributed) or Wilcoxon signed rank test (non-normally distributed). Categorical variables were compared using a chi-square test or McNemar test. Logistic regression analysis was performed to assess the independent contributions of individual confounding parameters on live birth in GH+cycle and multiple logistic regression analyses were performed to investigate the relationship between GH administration protocol and cumulative live birth using *p* < 0.10 of the likelihood ratio test and clinical consensus for inclusion. Statistical analyses were performed using SPSS version 26.0 (IBM, Armonk, NY, USA) and a two-tailed *P* < 0.05 was considered statistically significant.

## 3 Results

A total of 150 women aged 35-43 with both a GH-free cycle (GH−) and a subsequent GH administration cycle (GH+) were finally enrolled in the study. The demographic characteristics of the participants between the GH− and GH+cycle are summarized in **Table 1**. The age and duration of infertility between the paired cycles were statistically significant while the ovarian reserve parameters showed no significant difference.

**Table 1:** Baseline demographic characteristics of the participants in GH− and GH+ cycle.

	GH−	GH+	p-value
Age (years)	37.79 ± 2.39	38.33 ± 2.20	<0.001*
Infertility duration (years)	3 (2–5)	3 (2–5)	<0.001*
Basal FSH (IU/L)	9.01(7.35-10.92)	8.83(6.96-11.04)	0.856
AMH (ng/ml)	1.26(0.69-2.31)	1.24(0.69-2.28)	0.133
AFC (n)	6 (4–9)	6 (4–9)	0.376
Cause of infertility**, n (%)			
Diminished ovarian reserve	33(22%)		
Tubal factor	51(34%)		
Endometriosis	12(8%)		
Anovulation	15(10%)		
Male factor	41(27.33%)		
Unexplained infertility	21(14%)		

Continuous values presented as mean ± SD (normally distribution) or median (IQR) if not.

GH, growth hormone; GH−, GH-free cycle; GH+, GH administration cycle; FSH, follicle-stimulating hormone; AMH, anti-Mullerian hormone; AFC, antral follicle count. * p<0.05, statistically significant.

** Some participants have more than one factor.

Parameters of the ovarian stimulation between the two cycles are presented in **Table 2**. No significant differences in the COH protocol, total Gn dosage, Gn duration, E_2_ levels and endometrial thickness on hCG day were found between the two groups.

**Table 2:** Comparison of the ovarian stimulation parameters between the GH− and GH+ cycle.

	GH−	GH+	p-value
COH protocol, n (%)			0.521
GnRH antagonist	77(51.3)	70(46.7)	
GnRH agonist	12(8.0)	16(10.7)	
Mild stimulation	54(36.0)	52(34.7)	
Others	7(4.7)	12(8.0)	
Total Gn dosage (IU)	2400(1800-3000)	2475(1987.5-3150)	0.105
Gn duration (day)	9.16 ± 2.69	8.87 ± 2.08	0.199
E_2_ levels on hCG day (pg/ml)	1145.38(439.73-2203.00)	1022.85(513.73-2302.56)	0.799
Endometrial thickness on hCG day (mm)	9(7-11)	8.3(7-10)	0.607

Categorical values are presented as number (%). COH, controlled ovarian hyperstimulation; GnRH, gonadotropin-releasing hormone; Gn, gonadotropin; E_2_, estradiol; hCG, human chorionic gonadotropin.

The comparison of reproductive outcomes between the two groups are shown in **Tables 3**, **4**. The numbers of oocytes retrieved, MII oocytes, 2PNs, transferrable embryos and Day 3 good-quality embryos in GH+ significantly increased compared to the GH− cycle. The proportion of cycles with no transferrable embryos was significantly reduced in GH+ cycle compared with previous GH− cycle. GH co-treatment cycles showed significantly higher clinical pregnancy rates, live birth rates both in fresh embryo transfer and frozen-thawed embryo transfer cycle. As we can see, compared to previous GH− cycle failed in achieving a live birth, the cumulative live birth rate of the GH+ cycle reached 33.33% up to the moment of calculation under circumstance of a little more total number of embryos transferred (300 *vs.* 236) but much more remaining transferrable embryos (142 *vs.* 31).

**Table 3:** Comparison of embryo laboratory outcome between GH− and GH+ group.

	GH−	GH+	p-value
Number of oocytes retrieved (n)	5(3-8)	6(3-11)	<0.001
Number of MII oocytes (n)	4(3-7)	5(3-8)	0.001
Number of 2PN (n)	3(1-5)	4(2-6.5)	<0.001
Number of transferrable embryos (n)	2(1-2)	2.5(2-4)	<0.001
Number of Day 3 good-quality embryos (n)	0(0-1)	0(0-1)	0.001
Cycles with no transferrable embryos (%)	32(21.3) *	3(2.0) *	<0.001

All values presented as median (IQR) except * as number (%).

MII, Metaphase II; 2PN, two pronuclei.

**Table 4:** Comparison of clinical outcome between GH− and GH+ group.

	GH−	GH+	p-value
Fresh ET cycles (n)	33	48	
Biochemical pregnancy rate, n (%)	3/33 (9.10)	25/48(52.08)	<0.001
Clinical pregnancy rate	2/33 (6.06)	21/48(43.75)	<0.001
Miscarriage rate, n (%)	2/2(100)	7/21(33.33)	<0.001
Live birth rate, n (%)	0/33	14/48(29.17)	0.001
Frozen-thawed ET cycles (n)	108	133	
Biochemical pregnancy rate, n (%)	20/108(18.52)	60/133(45.11)	<0.001
Clinical pregnancy rate, n (%)	13/108(12.04)	51/133(38.35)	<0.001
Miscarriage rate, n (%)	13/13(100)	14/51(27.45)	0.05
Live birth rate, n (%)	0/108	36/133(27.07)	<0.001
Cumulative live birth rate per oocyte retrieval cycle, n (%)	0/150	50/150(33.33)	<0.001
Total number of transferrable embryos	267	442	
Total number of transferred embryos	236	300	
Total number of remaining transferrable embryos	31	142	

ET, embryo transfer.

We further stratified the participants into two groups based on whether the live birth was achieved in the GH supplementation cycle and explored the association between GH administration as well as some other clinical parameters (age, BMI, ovarian reserve, COH protocol) and cumulative live birth using logistic regression analysis, as shown in **Table 5**. Univariate analysis showed that GH administration protocol and ovarian reserve were significant predictors of the cumulative live birth while the mild stimulation protocol had marginal statistical significance). Multivariate analysis showed that dosage regimen of GH administration (4 IU QOD- 4 IU QD) was an independent factor of live birth after adjustment for relevant confounders. Use of GH prior to Gn stimulation and lasting until the hCG day seemed to achieve a higher successful live birth rate.

**Table 5:** Univariate and multivariate logistic regression analysis of the relationship between several clinical parameters and cumulative live birth in GH+ cycle.

	Univariate analysis		Multivariate analysis	
Variables	OR (95% CI)	*p*-value	OR (95% CI)	*p*-value
GH administration protocol				
Others	1	–	1	–
4 IU QOD-4 IU QD	2.312 (1.123-4.759)	0.023	2.355 (1.074-5.163)	0.032
COH protocol				
GnRH antagonist	1	–	1	–
GnRH agonist	2.314 (0.769-6.967)	0.136	1.820 (0.570-5.811)	0.312
Mild stimulation	0.483 (0.211-1.103)	0.084	0.639 (0.266-1.531)	0.315
Others	0.900 (0.246-3.289)	0.873	1.904 (0.439-8.250)	0.390
Age				
< 38	1	–	1	–
38-40	0.881 (0.420-1.848)	0.737	1.169 (0.524-2.609)	0.703
> 40	0.529 (0.184-1.522)	0.237	0.712 (0.226-2.247)	0.563
BMI				
18.50-24.99	1	–		
< 18.50	3.045 (0.485-19.120)	0.235		
25.00-29.99	1.015 (0.452-2.279)	0.971		
≥30	0.290 (0.034-2.456)	0.256		
Ovarian reserve				
NOR	1	–	1	–
DOR	0.427 (0.210-0.866)	0.018	0.496 (0.223-1.106)	0.086

Ovarian reserve was assessed with a comprehensive evaluation based on AMH, AFC and basal FSH levels of the two cycles by two experienced clinicians (24). Here, basically, at least two of the three following criteria were qualified: AMH < 1.1ng/ml; AFC < 5-7; basal FSH ≥ 10U/L.

BMI, body mass index; NOR, normal ovarian reserve; DOR, diminished ovarian reserve.

## 4 Discussion

The management of women with advanced age in assisted reproduction has always been a tough issue to tackle especially in those with deteriorating ovarian function. An increasing number of evidence supporting the use of GH in ART have been emerging both in laboratory and clinical setting since it was firstly applied into the adjuvant treatment in ovarian stimulation in 1980s. To date, the utilization of GH in ART mostly focused on the population of poor ovarian response or expected low-prognosis. There are still many controversies on the utilization of GH, such as the target population, underlying mechanism, administration protocol and so on. Our study expands the knowledge of the optimal GH administration protocol and appropriate population.

It was assumed that GH may contribute to higher success rate probably through increasing the number of mature oocytes and embryos (14) or improving embryo quality (12, 25, 26).

In this study, we investigated the effect of GH supplementation on assisted reproductive outcome in women aged between 35 and 43 irrespective of their ovarian reserve or response to ovarian stimulation. It was found that compared with previous failed GH – cycle, the number of oocytes retrieved, MII oocytes, 2PNs, transferrable embryos and good-quality embryos in GH+ significantly increased. Consistent with the results reported by Liu et al. (27), we presented a tolerable live birth rate in GH+ cycle in similar to that reported in aged women in their study (POSEIDON group 2: 28.33%; group 4: 24.07%). Besides, a cumulative live birth rate per cycle of 33.33% was achieved in the GH+ cycle when comparable number of embryos transferred and much more transferrable embryos remained, which may also suggest the underlying effect of GH on ameliorating embryo quality.

The dosage and duration of GH administration varied significantly across different studies. It seemed to undergo a trend transition from a large dose (8-24 IU per day) with short duration (usually less than two weeks) (10–12, 20, 28, 29) to a relatively low dose with longer duration (17, 19, 30–32). To the best of our knowledge, there were few studies designed to explore whether different dose and duration of GH supplementation resulted in difference on IVF outcome (26). Liu et al. firstly reported a better clinical outcome in women accepted GH with longer duration and higher dosage but the result of live birth was absent. We firstly reported that GH administration protocol was an independent factor enhancing the cumulative live birth per oocyte retrieval cycle in this study. GH dosage regimen of a long-term pretreatment at a relatively low dose prior to Gn stimulation and until hCG injection could optimize its efficacy. Similarly, a recent meta-analysis hypothesized there might be a dose- and time-dependent relationship between different GH protocols and IVF outcomes (33). Cai et al. (19) also reported 6-week pretreatment with GH could increase the live birth rate of poor ovarian responders in a retrospective self-controlled study.

A similar finding has been proposed in several studies that the administration of GH seems to exert less or no action in improving reproductive outcome in participants older than 40-42 years in the subgroup analysis stratified by age (31, 32, 34), It was inferred that may be attributed to the insufficient counteraction to GH deficiency at a dosage regimen of 2 IU for 42 days (31). The serum IGF-I levels were negatively correlated with age (35). The median serum IGF-I level was 239.7 ng/ml in healthy female adults aged 25-29 while that decreased to 186.0 ng/ml at the age of 35–39 and 167.2 at the age of 40-44. Follicular fluid (FF) GH and IGF-1 concentrations have been reported to be significantly lower in women having IVF who failed to become pregnant (36), and FF IGF-1, the effector of GH action, is reduced by fully one-half in poor responders (37). The age of the participants enrolled in our study was between 35 and 43, and there’s no significant difference found in further subgroup analysis by age. Interestingly, however, univariate logistic regression analysis showed that ovarian reserve was significant predictor of cumulative live birth while no significance was found after adjustment. It may remind us of the fact that growth hormone supplementation cannot counteract the decline in those with poor ovarian reserve while appropriate dosage regimen might offset in some extent. The same is true for the impact of age. Under this circumstance, whether the use of GH should be individualized rather than under the same dosage regimen. If so, whether the IGF or IGFBP level tests should also be enrolled in the ART treatment just as the routine examination of sex hormone during the Gn administration to guide clinical medication. Norbert et al. have ever proposed a similar opinion that the determination of IGF values may be indicated in women with low functional ovarian reserve (38).

Notably, researchers have also shed a new light on the role of GH in ameliorating blastocyst euploidy rates recently (31). Liu et al. also demonstrated GH supplementation not only alleviated decline in oocyte number and improved the quality, but also reduced aneuploidy in aged mice (38).

Consequently, given the consensus of decreased fertility with aging due to decline in the oocyte quantity and quality as well as rise in aneuploidy rate and reported effect of GH in ameliorating associated parameters above, an opinion was proposed, that is the use of GH supplementation in women with advanced maternal age might be supposed to take into consideration, not only in patients with diminished ovarian reserve or poor ovarian response but also in those expected normal prognosis. The evidence presented above shed a new light on reconsidering the appropriate timing of GH administration in the clinical practice. It might be of greater significance to use GH in clinical practice in advance when one’s fertility has not been too bad rather than taking it into consideration for making every possible effort. The age-related fertility decline are ignored or underestimated (39) by many practitioners. In fact, GH is underutilized in current clinical practice because of the “off-label” use and inertia and an inherent conservatism of many practitioners on adoption of new approaches (40).

There are also some limitations of the current study. First of all, it was truly the intrinsic limitations of a retrospective observational study. Secondly, it was a self-controlled study comparing outcome between the GH – and subsequent GH+ cycle but some clinical characteristics were not absolutely equal such as the status of the patients, stimulation protocols and so on. Besides, the sample size was not large enough so that some subgroup analyses were not available to further accomplish as expected.

In summary, administration of GH in women with advanced maternal age might be meaningful considering the findings of its effect on improvement in oocytes number, embryo quality and blastocyst euploidy rate. GH administration at a relatively low dose (2-4 IU per day) with longer duration (six-week or even longer) are of greater efficacy. Additionally, it’s worthy of exploring the necessity of GH-related tests and whether an individualized dosage regimen based on the level of GH, IGF or IGFBP of each patient would be more reasonable and effective. In the future, more well-designed prospective trials with large sample size and fundamental experiments on the mechanism are required to further testify the efficacy of GH and better guide its utilization in ART.

## Data availability statement

The raw data supporting the conclusions of this article will be made available by the authors, without undue reservation.

## Ethics statement

The studies involving humans were approved by Institutional review board (IRB) of Peking University Peoples’ Hospital. The studies were conducted in accordance with the local legislation and institutional requirements. The ethics committee/institutional review board waived the requirement of written informed consent for participation from the participants or the participants’ legal guardians/next of kin because all procedures were not altered from routine clinical practice, in accordance with the national legislation and the institutional requirements.

## Author contributions

QF: Data curation, Formal Analysis, Methodology, Software, Visualization, Writing - original draft. YW: Conceptualization, Funding acquisition, Supervision, Writing - review & editing. HH: Conceptualization, Funding acquisition, Methodology, Project administration, Writing - review & editing. HS: Conceptualization, Project administration, Supervision, Writing - review & editing.
